# The effect of sports participation on the social identity of Chinese university students

**DOI:** 10.3389/fpsyg.2024.1511807

**Published:** 2024-11-28

**Authors:** Shuyu Ji, Shuwei Chen, Xiannan Yang, Tongshang Liu, Xiaolin Wang

**Affiliations:** ^1^College of Physical Education and Sports, Beijing Normal University, Beijing, China; ^2^Institute of Athletic Training Science, Capital University of Physical Education and Sports, Beijing, China; ^3^Department of Sport Studies, Faculty of Educational Studies, University Putra Malaysia, Serdang, Malaysia

**Keywords:** sports participation, social identity, social mindset, Chinese, university students

## Abstract

**Objective:**

Strengthening youth sports and comprehensively promoting the participation of Chinese university students in sports to enhance their social acceptance are key initiatives for delivering high-quality talent for China’s economic and social development.

**Methods:**

Using the China Comprehensive Social Survey as the data source and considering social mentality and social identity, we constructed corresponding statistical models to explore how sports participation influences the social identity (class identity, economic status, and emotional belonging) of Chinese university students.

**Results:**

Regarded from a holistic perspective, sports participation can enhance the social identity of college students in all its aspects, and the benefits of sports participation are positively related to its frequency. Under the condition of social mentality, sports participation has a positive effect on the social identity of college students in general and a nonsignificant effect only on certain dimensions. Under the social identity condition, the significance of sports participation for college students’ social identity decreases from lower to higher levels of education.

**Conclusion:**

Sports participation significantly enhances the social identity of Chinese university students, and the specific mechanism of the enhancement varies across the social mentality and social identity conditions. In this context, strengthening the role of sports participation in promoting the social identity of college students in the new era is necessary. Deepening the ideological content of physical education courses should be taken as the starting point to enhance the effectiveness of sports participation on the social identity of college students under the condition of social mentality. The effectiveness of sports participation on the social identity of college students with different social identities should be differentiated and enhanced.

## 1 Introduction

Since the onset of the twenty-first century, China has entered a period of high-quality economic and social development and become more reliant on the social attributes of sports participation, a reliance that has also significantly improved the Human Development Index (HDI) of the Chinese people (Ji et al., 2023). Sports participation plays an important role in the development of the Chinese, whereby the increasing social recognition of talented athletes plays an important role in promoting high-quality economic and social development. Chinese university students (hereinafter referred to as university students) are an important component of China’s talent pool. Therefore, research focusing on university students’ sports activity and efforts to reinforce the positive effects of sports participation on university students’ social identity are significant. Because university students represent a reserve of talent for national economic and social development, their social identity involves their patriotism (Li et al., 2014) and orientation toward the socialist system with Chinese characteristics as well as toward the national governance system (Wu, 2020), which together comprise a key factor in determining their support for the socialist cause (Xie and Kong, 2022) and are particularly important for the sustainable development of the country’s economy and society. However, external forces frequently spread anti-socialist culture through diverse and covert means, confusing facts in an effort to sidetrack the social identity development orientation of college students and resulting in a serious impact on the social identity of Chinese university students (Chen, 2022). Improving the social identity of university students has become an urgent task.

Sports participation is an important means to promote the comprehensive development of human beings (Ji et al., 2023), is an important way to build social identity, can shape the collective consensus, and can provide psychological discipline to enable individuals to build a “self-ethics” in the “circle society” and guide university students in developing a positive social identity (Wang, 2020). However, previous studies have noted that the social identity of university students is influenced by both the “social mentality” and the “social identity.” The social mentality is influenced mainly by the trend of “paradoxical dialectics.” For example, ① the social mentality of university students reflects a conflation of Buddhism (in terms of unambitiously “lying flat” and being involuted) with social fear (accompanied by the explosion in internet use) and with political indifference and a susceptibility to political fervors. These circumstances create multiple dilemmas for university students in realizing their social values and seriously affect the orientation of social identity formation (Li, 2022). ② The orientation of social identity formation leads to a differentiated social mentality among university students. Overall, this mentality can be categorized as including policy preferences such as placing one’s country before one’s family, but also negative social evaluations and populist tendencies as well as diverse views regarding social order and national security (Zheng et al., 2023). ③ The social mindset brings a “threshold experience” to university students’ sport participation, reinforcing the positive effects of social responsibility and social justice on the social identity of such students (Wang, 2020). Social identity is largely influenced by questions such as “Who do they want me to be?” or “Who do I want me to be?” ① Cruess et al. (2015) noted that higher education must help university students carry out a step-by-step transformation of their social identity and, in the process of transformation, consolidate a social identity oriented toward socialist core values. ② Higher education should also assist university students clarify their social identities while strengthening the social position of individuals to promote the adoption of socially appropriate behavioral measures while gradually enhancing social identity (Wilson and Liss, 2022). ③ The social identity of university students significantly influences the frequency of sports participation through the mediating factor of sports culture, which in turn consolidates social identity and social identification (Gonzales et al., 2020; Zheng and Liu, 2013; Cruess et al., 2015). ④ Dislocation of the social identity of university students will lead to a serious deviation from the “expected structure” and “real structure” of social identity, placing students in the embarrassing situation of double discrimination in economic status and access qualification (Zheng and Liu, 2013; Hoyt et al., 2021). Previous studies provide diverse theories, methods, and perspectives for subsequent research. However, the mechanism influencing university students’ social identity and how to promote students’ social identity from the perspective of sports participation have rarely been examined.

Therefore, this study utilized data from the China General Social Survey (CGSS) released by the China Survey and Data Center in October 2022 for the construction of several statistical models to explore the effectiveness of sports participation on the social identity of Chinese university students and the mechanisms of social mentality and social identity formation. The study also provides references and lessons for further enhancing the social identity of Chinese university students and promoting their overall development.

## 2 Research design

### 2.1 Data sources, sample selection, indicator selection

#### 2.1.1 Data sources

The data for this study were drawn from the China General Social Survey (CGSS) data released in October 2022 by Renmin University of China and the China Survey and Data Center. This database comprehensively and systematically collects data at multiple levels, including the social, family, and individual levels, and summarizes trends in social change. It has been used in the East Asian Social Survey Project (EASS), which aims to provide reliable data support for a wide range of researchers.

#### 2.1.2 Sample selection

The total number of variables in the database for 2022 was 1,030, and the total sample size was 12,787. Samples in the disability category were excluded, samples other than specialty, undergraduate, and postgraduate students were excluded, and non-target variables were excluded. After excluding nontarget variables and missing values, the number of target variables selected was 12, leaving a valid sample of 2,320 data points.

#### 2.1.3 Indicator selection

① Social identity (dependent variable): International scholars mainly examine social identity through social comparison and psycho-emotions, social inequality, social status, wealth creation ability, economic strength, self-perception, emotional expression, and other indicators (Anyiwo et al., 2018; Yang et al., 2018; Hehir et al., 2021). Chinese scholars, in contrast, tend to investigate social identity with indicators such as social class perception, emotional identity, consumption concepts, membership, and role emotions (Peng, 2012; Xiao, 2015; Leng et al., 2021). In view of this, this study synthesizes the views of these groups and uses three dimensions, namely, class identity, economic status, and emotional belonging, as reflective indicators of social identity among Chinese university students. ② Social mentality and social identity (independent variables): The relevant academic views on social mentality (Wang, 2020; Li, 2022; Zheng et al., 2023) and social identity (Gonzales et al., 2020; Zheng and Liu, 2013; Cruess et al., 2015; Hoyt et al., 2021) have been described above. Therefore, considering the indicators used in previous studies and the characteristics of the university student group, social trust and social fairness are used to reflect social mentality, and school level and academic level are used to reflect social identity. ③ Control variables: Based on relevant studies (Dong, 2021; Ji et al., 2023), household registration, gender, physical health, and mental health were used as reflective indicators.

### 2.2 Model setup

Pinar et al. (2013) noted that when multivariate, multidimensional, or multilevel methods reflect the same issue or the same indicator, there is a disparity in weighting between the internal indicators, which should be counted separately. Qiu et al. (2018) noted that when multiple variables reflect common indicators, they should be discussed separately rather than analyzed uniformly after weighted fitting if the nature of the problem is to be explored carefully. In view of this, this study takes three dimensions of the social identity of university students, namely, class identity, economic status, and emotional belonging, as dependent variables to reflect the social identity of university students in a detailed and objective manner.

The variable relationships explored in this study are between a dependent variable and multiple independent variables. Thus, multiple linear regression models are applicable. Moreover, the sample size for this study was 2,320, the number of variables was 12, and the ratio between the total sample size and the total variables was approximately 193:1, which circumvents the problem of multiple covariance in the multiple linear regression model; i.e., the ratio of the total sample size to the total variables was greater than 5:1. On this basis, models are constructed as follows:


(1) $$Y_{i}=\mathrm{LnOSpor}t_{i}+X_{i}+\varepsilon_{i}$$

This is Equation (1) (sports participation), where Y is the dependent variable (social identity), i represents different dimensions of social identity (class identity, economic status, and emotional belonging), $\mathrm{LnOSpor}t_{i}$ is the core explanatory variable (sports participation), $X_{i}$ represents control variables (such as demographic characteristics), and $\varepsilon_{i}$ is a constant term.


(2) $$Y_{i}=\mathrm{LnOSpor}t_{i}+\mathrm{InMindse}t_{i.j}+X_{i}+\varepsilon_{i}$$

This is Equation (2) (social mindset), where InMindset is the social mindset, j is the variable included under the social mindset.


(3) $$Y_{i}=\mathrm{LnOSpor}t_{i}+\mathrm{InIdentit}y_{i.k}+X_{i}+\varepsilon_{i}$$

This is Equation (3) (social identity), where InIdentity is social identity, k is the variable included under social identity.

To explore in depth the specific impact of social mentality and social identity, the two variables are used as categorical variables to build a regression model, and at the same time, to ensure that the evidence derived from the categorical variable model is in line with the real situation. If one of the two variables is used as a categorical variable, the other variable will still be included in the statistics.

Models 4–9 explore social mindfulness as a categorical variable as follows:


(4) $$Y_{i.j}=\mathrm{LnOSpor}t_{i.j}+\mathrm{InMindse}t_{i.k.-j}+\mathrm{InIdentit}y_{i.k.j}+X_{i.j}+\varepsilon_{i.j}$$

Models 10–15 explore social identity as a categorical variable as follows:


(5) $$Y_{i.k}=\mathrm{LnOSpor}t_{i.k}+\mathrm{InIdentit}y_{i.j.-k}+\mathrm{InMindse}t_{i.j.k}+X_{i.k}+\varepsilon_{i.k}$$

In Equations 4, 5, −j and −k represent specific variables that have not yet been used as categorical variables in the social mindset and social identity models, respectively. Because the control variables have been controlled for according to previous research on model construction (Qiu et al., 2018), these variables are not presented in an expanded manner.

### 2.3 Variable manipulation and description

#### 2.3.1 Dependent variable (social identity)

① Class identity: This variable aims to investigate at which level of the subject’s own present social class. The CGSS investigates class identity by positively coding the ranks on a scale of 1 to 10, with 1 being the lowest and 10 being the highest. ② Economic status: As one of the core variables, economic status aims to investigate the economic status of the subjects in present society. The CGSS surveyed economic status at 5 levels, lower, lower middle, middle, upper middle, and upper, reverse coded. For statistical convenience, the 5-level division was followed, but the coding was adjusted to be forward coded. ③ Affective attributions: Affective attributions were positively coded by asking the subjects about the degree of happiness they experienced in the current society and their feelings of dependence and belonging to the current society, categorized as low, medium, or high.

#### 2.3.2 Impact core

Physical participation. Moving one’s body until one is perspiring through activities such as walking, running, physical training, square dancing, radio gymnastics, and ball games. The exercise format may be freely chosen, the participation forms could be individual, group or combination activity. From above, it was to investigate the frequency of sports participation in the past year in subjects with a combination of the above conditions. The sports participation variables were reverse coded with full reference to the categorization criteria used in the CGSS survey, i.e., daily, weekly, monthly, and never.

#### 2.3.3 Control variables

① The household registration variable includes the agricultural (rural) type sample of former agricultural (rural) households that became family households; the sample of former nonagricultural (urban) households that became family households is included in the nonagricultural (urban) type. ② There are only two categories of gender: male and female. ③ Physical health and mental health were positively coded as unhealthy, fair, and healthy.

#### 2.3.4 Independent variables

① Academic level was categorized as specialist, undergraduate or postgraduate after nontarget samples were excluded and positively coded. School level was categorized as ministry and above or province and other, positively coded, with a special note: joint colleges and universities were categorized as the higher level; e.g., province–ministry joint colleges were categorized as ministry-level, and province–municipality joint colleges were categorized as province-level (Li et al., 2022). ② In the dimension of social mentality, social trust examines the degree of trust that subjects have in modern Chinese society, and social fairness measures the degree to which the subjects believe that modern Chinese society is fair to them. These variables are positively coded using a three-level scale (see Table 1) for details.

**Table 1:** Descriptive statistics of the sample.

Variable dimension	Variable and number	Coding method	Mean (SD)	Causality
Implicit variable	Class identity (CI) A34a	1 ~ 10	4.81 (1.48)	+
	Economic status (ES) A43e	below =1; middle and lower =2; middle =3; upper middle =4; first =5	2.56 (0.81)	+
	Affective attributions (AA) A36	lower =1; middle =2; high =3	2.81 (0.46)	+
Impact core	Sports participation (SP) A309	every day =1; every week = 2; every month =3; every year =4; no =5	2.69 (1.31)	−
Control variable (CV)	Sex A2	male =1; female =2	1.47 (0.49)	classify
	Household registration (HR) A18	Agriculture (rural) = 1; Nonagricultural (urban) =2	1.71 (0.45)	classify
	Wellness (WL) A15	no =1; middle =2; yes =3	2.67 (0.59)	+
	Mental health (MH) A16	no =1; middle =2; yes =3	2.69 (0.55)	+
Social mentality	Social trust (ST) A33	no =1; middle =2; yes =3	2.51 (0.77)	+/classify
	Social equity (SE) A35	no =1; middle =2; yes =3	2.22 (0.83)	+/classify
Social identity	School level (SL) A7e	other =1; province =2; ministry =3	2.40 (0.72)	+/classify
	Education level (EL) A7a	specialty =1; undergraduate =2; postgraduates =3	1.66 (0.61)	+/classify

#### 2.3.5 Descriptive statistics of correlations between variables

In order to investigate the correlation between various variables and enhance the statistical efficiency of the regression model, a correlation test was specifically conducted. Overall, as it can be seen from Table 2, there is a strong correlation between most of variables, however the correlation between only some of the variables is weak. Upon closer inspection, the impact core (SP) shows no correlation with ST, but has a strong correlation with other variables. The correlation between the implicit variables (CI, ES, AA) and other variables is also relatively strong, indicating that the variables used in this study for regression analysis have high statistical efficiency.

**Table 2:** Correlation analysis.

	CI	ES	AA	Sex	HR	WL	MH	SP	ST	SE	SL	EL
CI	1.000											
ES	0.644***	1.000										
AA	0.150***	0.176***	1.000									
Sex	0.028	0.013	−0.010	1.000								
HR	0.106***	0.130***	−0.018	−0.011	1.000							
WL	0.081***	0.072***	0.177***	0.005	−0.109***	1.000						
MH	0.150***	0.151***	0.246***	−0.045**	0.027	0.287***	1.000					
SP	−0.113***	−0.083***	−0.120***	−0.109***	−0.044**	−0.132***	−0.128***	1.000				
ST	0.104***	0.107***	0.216***	−0.005	0.015	0.047**	0.096***	−0.033	1.000			
SE	0.173***	0.165***	0.243***	0.003	0.015	0.093***	0.145***	0.065***	0.065***	1.000		
SL	−0.088***	−0.093***	−0.019	0.048**	−0.092***	0.023	0.011	0.066***	−0.057***	−0.016	1.000	
EL	0.112***	0.083***	−0.004	−0.024	0.085***	−0.012	−0.004	−0.059***	0.013	0.039*	−0.405***	1.000

“+” denotes *p* < 0.1, “*” denotes *p* < 0.05, “**” denotes *p* < 0.01, “***” denotes *p* < 0.001; “0” is omitted when the single digit is “0”.

## 3 Empirical analysis and discussion

### 3.1 Effect of sports participation on social identity among Chinese university students

According to Table 3, sports participation (*p* < 0.05) significantly contributes to the social identity of university students and is not significantly affected by social mentality or social identity. The mechanism of action is manifested in the fact that the greater the frequency of sports participation is, the greater the increase in social identity, which is inversely due to the negative factorial coding of the sports participation variable. Model 2 (social mentality) shows that under the influence of sports participation, social trust (*p* < 0.01) and social equity (*p* < 0.001) in social mentality can positively affect the social identity of university students, which is manifested in the fact that the higher the degree of social trust and social equity, the higher their social identity. Model 3 (social identity) shows that, under the influence of sports participation, school level promotes the economic status (*p* < 0.01) of university students more strongly and promotes class identity (*p* < 0.05) more weakly, and its promotion effect is as follows: Ministry >Provincial>Other. In addition, school level has no significant effect on affective attributions. Education level significantly enhances the class identity (*p* < 0.001) and economic status (*p* < 0.05) of university students, and its effect benefit is as follows: Graduate school>Undergraduate school>Specialized school. There is no significant effect on affective attributions. Based on the value of the explanatory rate R^2^ and compared with Model 1 (CI = 0.046; ES = 0.046; AA = 0.079), social mentality (CI = 0.070; ES = 0.068; AA = 0.140) and social identity (CI = 0.058; ES = 0.054; AA = 0.079) exert a certain facilitating effect at the same time, and the *R*^2^ value of class identity, economic status and affective attributions displayed alternating changes, indicating that the three dimensions of the social identity of university students have different weight shares, verifying the views of Pinar et al. (2013) and Qiu et al. (2018), subsequent studies should explore these issues separately.

**Table 3:** Effects of sport participation on the social identity of Chinese university students.

Variant	Model 1 (Sports participation)			Model 2 (Social mentality)			Model 3 (Social identity)		
	CI	ES	AA	CI	ES	AA	CI	ES	AA
Sex	0.132 (0.060)*	0.043 (0.033)	0.006 (0.018)	0.129 (0.059)*	0.041 (0.033)	0.005 (0.018)	0.142 (0.060)*	0.048 (0.033)	0.007 (0.018)
HR	0.361 (0.066)***	0.245 (0.036)***	−0.004 (0.020)	0.348 (0.066)***	0.238 (0.036)***	−0.011 (0.020)	0.326 (0.066)***	0.229 (0.036)***	−0.005 (0.020)
WL	0.109 (0.053)*	0.054 (0.029)^+^	0.084 (0.016)***	0.088 (0.052)^+^	0.043 (0.029)	0.074 (0.016)***	0.112 (0.053)*	0.056 (0.029)^+^	0.084 (0.016)***
MH	0.347 (0.056)***	0.201 (0.031)***	0.172 (0.017)***	0.290 (0.056)***	0.170 (0.031)***	0.143 (0.017)***	0.351 (0.056)***	0.203 (0.031)***	0.172 (0.017)***
SP	−0.102 (0.023)***	−0.035 (0.012)**	−0.028 (0.007)***	−0.095 (0.023)***	−0.031 (0.012)*	−0.025 (0.007)***	−0.094 (0.023)***	−0.031 (0.012)*	−0.028 (0.007)***
ST				0.105 (0.039) **	0.062 (0.021)**	0.091 (0.012)***			
SE				0.235 (0.036)***	0.121 (0.020)***	0.093 (0.011)***			
SL							0.091 (0.045)*	0.070 (0.024)**	0.016 (0.014)
EL							0.197 (0.052)***	0.060 (0.029)*	−0.012 (0.016)
con-	3.538 (0.212)***	1.779 (0.117)	2.205 (0.065)***	2.949 (0.228)***	1.456 (0.126)***	1.866 (0.069)***	3.409 (0.265) ***	1.837 (0.147)***	2.265 (0.083)***
N	2,320	2,320	2,320	2,320	2,320	2,320	2,320	2,320	2,320
*R*^2^	0.046	0.046	0.079	0.070	0.068	0.140	0.058	0.054	0.079

Standard errors are shown in parentheses.

Models 2–3 are based on Model 1 with the addition of social mentality and social identity, respectively, while the significant degree and role benefit of sports participation (*p* < 0.05) remain unchanged, indicating that the impact of sports participation on the social identity of university students has strong stability and can still have a facilitating effect under the influence of comprehensive factors. Yu and Zuo (2023) noted that the media era has prompted university students’ sports participation to shift from health-seeking to “persona” shaping, which includes searching for affective attributions, confirming spending power, and upwardly integrating into class identity. Model 2 shows that social mentality (*p* < 0.01) has a positive moderating effect, which enhances the promotional benefits of sports participation on university students’ social identity. Sun and Zheng (2017) argued that a Chinese Red Culture-guided social mentality enhances political certainty, economic orientation, cultural self-confidence, and emotional cognition and that integrating such a mentality into the sports curriculum ideology can enhance university students’ social identity. Model 3 shows that social identity positively moderates class identity (*p* < 0.05) and economic status (*p* < 0.05) but has no significant effect on affective attributions, for the following possible reasons. ① Justice education, which emphasizes social identity, encourages individual students to clarify their class and position in the social group, while sports participation facilitates the progression from holding a “justice view” to being a “social justice practitioner” (Qin, 2023). ② The rise of online social networking has led to false fulfillment of the social desires of university students, and the absence of identity differences has limited the cognitive level of university students and weakened their ability to interact; however, a unique affective attribution has occurred such that the crisis of generalized online social networking has intensified and seriously inhibited the sound development of the affective attributions of university students (Chen, 2023).

### 3.2 Effects of different social mentalities in sports participation on the social identity of Chinese university students

Table 4 presents results for several models. Model 4 for concerns university students and social trust. Sports participation (*p* < 0.01) contributed more to affective attributions and but less to class identity (*p* < 0.1) and economic status (*p* < 0.1). Under the influence of sports participation, social equity was a strong contributor to economic status (*p* < 0.05) and affective attributions (*p* < 0.001), with no significant effect on class identity; school level was a significant contributor to economic status (*p* < 0.05), with no significant effect on class identity and affective attributions; and education level was a weakly significant contributor to affective attributions (*p* < 0.1), with no significant effect on class identity and economic status. Model 5 concerns university students who are neutral about social trust. Sports participation had a significant contributing effect on class identity (*p* < 0.05) and economic status (*p* < 0.05) but had no significant effect on affective attributions. Social equity made a stronger contribution to class identity (*p* < 0.05) and affective attributions (*p* < 0.05) but made a lower contribution to economic status (*p* < 0.1) under the influence of sports participation; school level and education level did not have significant effects. Model 6 is for university students who have social trust. Sports participation made a strong contribution to class identity (*p* < 0.05) and affective attributions (*p* < 0.05) but had no significant effect on economic status. Under the influence of sports participation, social equity (*p* < 0.001) had a comprehensive facilitating effect on the social identity of this type of university student; school level had a weakly significant facilitating effect on class identity (*p* < 0.1) and economic status (*p* < 0.1) but no significant effect on affective attributions; education level had a significant facilitating effect on class identity (*p* < 0.01) and economic status (*p* < 0.05) but no effect on affective attributions.

**Table 4:** Effects of different social mentalities in sports participation on the social identity of Chinese university students.

Variant	Model 4 (Social trust-no)			Model 5 (Social trust-middle)			Model 6 (Social trust-yes)		
	CI	ES	AA	CI	ES	AA	CI	ES	AA
Sex	0.203 (0.159)	0.142 (0.082)^+^	−0.025 (0.059)	0.135 (0.168)	0.006 (0.091)	−0.089 (0.059)	0.122 (0.069)^+^	0.032 (0.039)	0.030 (0.018)^+^
HR	0.386 (0.170)*	0.239 (0.088)**	0.051 (0.063)	0.340 (0.190)^+^	0.129 (0.103)	0.027 (0.067)	0.299 (0.077)***	0.243 (0.043)***	−0.034 (0.020)^+^
WL	0.125 (0.125)	0.024 (0.064)	0.047 (0.046)	0.141 (0.158)	0.063 (0.086)	0.212 (0.056)***	0.073 (0.062)	0.046 (0.035)	0.062 (0.016)***
MH	0.366 (0.126)**	0.285 (0.065)***	0.212 (0.047)***	0.312 (0.154)*	0.054 (0.083)	0.189 (0.054)***	0.263 (0.069)***	0.159 (0.039)***	0.102 (0.017)
SP	−0.099 (0.059)^+^	−0.058 (0.030)^+^	−0.065 (0.022)**	−0.163 (0.067)*	−0.082 (0.036)*	−0.035 (0.024)	−0.069 (0.027)*	−0.010 (0.015)	−0.016 (0.007)*
SE	0.114 (0.088)	0.101 (0.046)*	0.187 (0.033)***	0.259 (0.113)*	0.120 (0.061)^+^	0.094 (0.040)*	0.263 (0.043)***	0.127 (0.024)***	0.070 (0.011)***
SL	0.080 (0.119)	0.156 (0.062)*	−0.022 (0.044)	0.034 (0.137)	0.044 (0.074)	0.063 (0.048)	0.088 (0.051)^+^	0.048 (0.029)^+^	0.004 (0.013)
EL	0.223 (0.137)	−0.012 (0.071)	−0.100 (0.051)^+^	0.033 (0.148)	0.020 (0.080)	0.013 (0.052)	0.208 (0.061)**	0.080 (0.034)*	0.005 (0.015)
con-	2.802 (0.670)***	1.751 (0.348)***	1.891 (0.252)***	3.033 (0.790)***	2.125 (0.429)***	1.695 (0.279)***	3.155 (0.318)***	1.584 (0.179)***	2.326 (0.082)***
N	409	409	409	304	304	304	1,607	1,607	1,607
*R*^2^	0.066	0.108	0.166	0.080	0.046	0.165	0.073	0.065	0.083

	Model 7 (Social equity-no)			Model 8 (Social equity-middle)			Model 9 (Social equity-yes)		
	CI	ES	AA	CI	ES	AA	CI	ES	AA
Sex	0.203 (0.119)^+^	0.114 (0.063)^+^	−0.025 (0.048)	0.216 (0.124)^+^	0.089 (0.069)	0.018 (0.038)	0.084 (0.082)	0.003 (0.046)	0.019 (0.018)
HR	0.429 (0.131)***	0.196 (0.070)**	−0.010 (0.052)	0.174 (0.139)	0.151 (0.078)^+^	−0.001 (0.042)	0.338 (0.091)***	0.283 (0.051)***	−0.011 (0.020)
WL	0.156 (0.095)	0.061 (0.051)	0.121 (0.038)**	0.158 (0.110)	0.081 (0.061)	0.100 (0.033)**	0.008 (0.077)	0.006 (0.043)	0.029 (0.017)^+^
MH	0.401 (0.095)***	0.270 (0.051)***	0.181 (0.038)***	0.177 (0.113)	0.045 (0.063)	0.108 (0.034)**	0.283 (0.088)***	0.171 (0.049)***	0.135 (0.019)***
SP	−0.083 (0.045)^+^	−0.037 (0.024)	−0.040 (0.018)*	−0.116 (0.049)*	−0.053 (0.027)^+^	−0.038 (0.015)**	−0.070 (0.032)*	−0.011 (0.018)	−0.012 (0.007)^+^
ST	0.065 (0.065)	0.069 (0.034)*	0.155 (0.026)***	0.020 (0.083)	0.008 (0.046)	0.061 (0.025)**	0.188 (0.062)**	0.083 (0.034)*	0.048 (0.014)***
SL	−0.024 (0.086)	0.047 (0.046)	−0.013 (0.034)	0.269 (0.093)**	0.157 (0.052)**	0.033 (0.028)	0.052 (0.062)	0.030 (0.035)	0.010 (0.014)
EL	0.310 (0.099)**	0.141 (0.053)**	−0.002 (0.040)	−0.005 (0.109)	−0.017 (0.061)	−0.045 (0.033)	0.215 (0.074)**	0.040 (0.041)	−0.007 (0.016)
con-	2.159 (0.513)***	1.173 (0.274)***	1.622 (0.207)***	4.478 (0.598)***	2.542 (0.334)***	2.341 (0.183)	3.413 (0.414)***	1.808 (0.232)***	2.402 (0.093)***
*N*	612	612	612	565	565	565	1,143	1,143	1,143
*R* ^2^	0.087	0.101	0.131	0.047	0.040	0.074	0.051	0.048	0.074

Model 7 addresses socially unequal university students. Here, sports participation made a significant contribution to affective attributions (*p* < 0.05) and a weakly significant contribution to class identity (*p* < 0.1) but had no significant effect on economic status. Under the influence of sports participation, social trust made a significant contribution to economic status (*p* < 0.05) and affective attributions (*p* < 0.001) but exerted no significant effect on class identity; school level had no significant effect; and education level made a significant contribution to class identity (*p* < 0.01) and economic status (*p* < 0.01) but had no significant effect on affective attributions. Model 8 concerns social equity-neutral university students. Here, sports participation had a strong facilitating effect on class identity (*p* < 0.05) and affective attributions (*p* < 0.01) and a weak facilitating effect on economic status (*p* < 0.1). Under the influence of sports participation, social trust exerted a significant contributing effect on affective attributions (*p* < 0.01) but no significant effect on class identity and economic status; school level had a significant contributing effect on class identity (*p* < 0.01) and economic status (*p* < 0.01) but no significant effect on affective attributions for this type of university student; and education level had no significant effect. Model 9 addresses university students with social. Sports participation exerted a significant contributing effect on class identity (*p* < 0.05) and a weak facilitating effect on affective attributions (*p* < 0.1), but had no significant effect on economic status. Under the influence of sports participation, social trust (*p* < 0.05) could enhance the social identity of this type of university student in all aspects; school level had no significant effect; education level had a significant contributing effect on class identity (*p* < 0.01) but no significant effect on economic status or affective attributions.

Sports participation had no significant effect on the affective attributions of social trust-neutral university students or on the economic status of the three types of university students: social trust, social inequity, and social equity. The remaining social mentality conditions showed a degree of significant facilitation, with only certain dimensions exhibiting relatively weak facilitation. The reasons for this are as follows. ① Ouyang et al. (2017) confirmed that the dual functions of social–emotional and event attachment under differential social trust significantly inhibited the degree of identification with sports tourism and failed to enhance the frequency of sports participation in a neutral-level trust group, whereas enhancing governmental trust could modulate the strength of support for the emotional aspect of sports participation. Social mentality only partially explains the relationship between economic status and athletic health (Wang and Ma, 2020), and during the period of the mass spread of higher education, the noneconomic effect of social trust is greater than the economic effect (Zhou, 2016); therefore, under the social mentality condition, sports participation only has a strong facilitating effect on the economic status of certain groups of university students. In the social mentality condition, the facilitating effect of sports participation on the social identity of university students was weakly supported by the aspect of social identity, with only certain dimensions and variables providing significant support. The possible reasons for this are investigated as follows. Wang and Chen (2024) noted that the current system of ideological education in colleges and universities has been unable to meet the needs of the times with respect to cultivating students’ virtues, and although research on the ideological aspects of physical education courses has been popular, the summarization and refinement of the endogenous elements and exogenous resources of public physical education courses from the perspective of course ideology has been rare. These circumstances have led to a lack of clarity regarding how to effectively combine physical education courses at Chinese colleges and universities with ideological education and made it difficult to promote the macrolevel of the social identity of college students. It is difficult to promote the overall improvement of the social identity of university students at the macrolevel. University students with different social identities have problems such as the deviation of some groups’ cognition and practice of socialist core values, a tendency toward individualization of value choice, and the formalization of value understanding, and the resolution of such problems urgently requires the integration of socialist core values into university education, student services and campus activities to enhance student social identities (Qu et al., 2021).

### 3.3 Effects of different social identities in sports participation on the social identities of Chinese university students

Table 5 presents results for a new set of models. Model 10 addresses university students at ministry-level and above universities. Sports participation and education level did not significantly affect the social identity of this type of university student. Social trust made a significant contribution to affective attributions (*p* < 0.001) but had no significant effect on class identity and economic status. Social equity (*p* < 0.01) could promote the social identity of this type of university student in all aspects. Model 11 concerns university students at provincial universities. There was no effect of education level. Sports participation had a significant contributing effect on class identity (*p* < 0.001) but no significant effect on economic status and affective attributions. Under the influence of sports participation, social trust had a significant contributing effect on affective attributions (*p* < 0.001) but no significant effect on class identity and economic status. Social equity exerted a significant contributing effect on class identity (*p* < 0.001) and affective attributions (*p* < 0.001) but no significant effect on economic status. School level had a weakly significant contributing effect on class identity (*p* < 0.1) but no significant effect on economic status or affective attributions. Model 12 addresses university students from other universities. Sports participation (*p* < 0.05) significantly promoted the social identity of this type of university student. Under the influence of sports participation, social trust (*p* < 0.01) and social equity (*p* < 0.001) could promote the social identity of this type of university student in all aspects, and only social trust had a weakly significant promoting effect on class identity (*p* < 0.1). Education level had a stronger promoting effect on class identity (*p* < 0.01), a weaker promoting effect on economic status (*p* < 0.1), and no significant effect on affective attributions.

**Table 5:** Effects of different social identities in sports participation on the social identity of Chinese university students.

Variant	Model 10 (School level ministry and above)			Model 11 (School level provincial)			Model 12 (School level other)		
	CI	ES	AA	CI	ES	AA	CI	ES	AA
Sex	−0.155 (0.157)	−0.008 (0.085)	0.001 (0.047)	0.095 (0.106)	−0.030 (0.062)	−0.015 (0.030)	0.226 (0.081)**	0.095 (0.043)*	0.014 (0.025)
HR	0.041 (0.202)	0.148 (0.110)	−0.079 (0.060)	0.429 (0.115)***	0.443 (0.067)***	0.033 (0.033)	0.286 (0.088)***	0.121 (0.047)**	−0.021 (0.027)
WL	−0.059 (0.124)	0.048 (0.068)	0.093 (0.037)*	−0.076 (0.099)	−0.054 (0.058)	0.086 (0.028)**	0.219 (0.071)**	0.085 (0.038)*	0.062 (0.022)**
MH	0.428 (0.136)**	0.281 (0.074)***	0.192 (0.041)***	0.317 (0.101)**	0.228 (0.059)***	0.109 (0.029)***	0.253 (0.076)***	0.116 (0.041)**	0.144 (0.024)***
SP	0.043 (0.064)	−0.005 (0.035)	−0.022 (0.019)	−0.140 (0.042)***	−0.024 (0.024)	−0.011 (0.012)	−0.086 (0.030)**	−0.032 (0.016)*	−0.032 (0.009)***
ST	0.136 (0.104)	0.028 (0.056)	0.121 (0.031)***	0.077 (0.073)	0.050 (0.043)	0.096 (0.021)***	0.103 (0.052)^+^	0.074 (0.028)**	0.084 (0.016)***
SE	0.252 (0.093)**	0.140 (0.051)**	0.078 (0.028)**	0.250 (0.066)***	0.050 (0.038)	0.111 (0.019)***	0.212 (0.050)***	0.148 (0.026)***	0.087 (0.015)***
EL	0.113 (0.131)	0.059 (0.071)	−0.035 (0.039)	0.196 (0.100)^+^	0.031 (0.058)	−0.010 (0.029)	0.218 (0.070)**	0.069 (0.037)^+^	−0.014 (0.022)
con-	2.901 (0.592)***	1.261 (0.323)***	1.763 (0.178)***	3.068 (0.472)***	1.567 (0.276)***	1.847 (0.136)***	2.341 (0.326)***	1.319 (0.174)***	1.962 (0.103)***
N	338	338	338	705	705	705	1,277	1,277	1,277
*R* ^2^	0.074	0.092	0.194	0.094	0.093	0.158	0.078	0.073	0.125

	Model 13 (Education level specialized)			Model 14 (Bachelor’s degree at education level)			Model 15 (Education level graduate)		
	CI	ES	AA	CI	ES	AA	CI	ES	AA
Sex	0.217 (0.095)*	0.088 (0.050)^+^	0.005 (0.028)	0.110 (0.081)	0.018 (0.046)	0.021 (0.024)	−0.008 (0.217)	0.079 (0.119)	−0.058 (0.067)
HR	0.196 (0.103)^+^	0.099 (0.054)^+^	−0.037 (0.031)	0.501 (0.090)***	0.357 (0.052)***	0.021 (0.027)	−0.212 (0.277)	0.034 (0.152)	−0.046 (0.085)
WL	0.158 (0.084)^+^	0.092 (0.044)*	0.105 (0.025)***	0.079 (0.072)	0.032 (0.041)	0.073 (0.022)***	−0.201 (0.186)	−0.107 (0.102)	−0.091 (0.057)
MH	0.350 (0.088)***	0.188 (0.046)***	0.109 (0.026)***	0.295 (0.077)***	0.183 (0.044)***	0.159 (0.023)***	0.098 (0.203)	0.036 (0.112)	0.211 (0.062)***
SP	−0.094 (0.034)**	−0.043 (0.018)*	−0.027 (0.010)**	−0.095 (0.032)**	−0.024 (0.018)	−0.024 (0.010)*	−0.033 (0.094)	0.001 (0.051)	−0.046 (0.029)
ST	0.041 (0.062)	0.015 (0.033)	0.080 (0.018)***	0.180 (0.053)***	0.096 (0.031)**	0.071 (0.016)***	−0.047 (0.147)	0.075 (0.081)	0.268 (0.045)***
SE	0.229 (0.058)***	0.161 (0.030)***	0.086 (0.017)***	0.246 (0.050)***	0.095 (0.029)***	0.118 (0.015)***	0.165 (0.135)	0.073 (0.074)	−0.027 (0.041)
SL	0.132 (0.089)	0.099 (0.047)*	0.011 (0.026)	0.062 (0.055)	−0.052 (0.031)	0.016 (0.016)	0.164 (0.134)	0.127 (0.074)^+^	0.001 (0.041)
con-	3.048 (0.436)***	1.641 (0.230)***	1.977 (0.131)***	2.851 (0.331)***	1.456 (0.190)***	1.822 (0.101)***	5.748 (0.870)***	2.756 (0.479)***	2.020 (0.269)***
N	965	965	965	1,169	1,169	1,169	186	186	186
*R* ^2^	0.071	0.085	0.126	0.094	0.085	0.160	0.026	0.042	0.266

Model (13) concerns university students with a specialized degree. Sports participation (*p* < 0.05) could promote the social identity of this type of university student in all aspects. Under the influence of sports participation, social trust had a significant positive effect on affective attributions (*p* < 0.001) but no significant effect on class identity and economic status. Social equity (*p* < 0.001) promoted the social identity of this type of university student in all aspects, and school level had a significant positive effect on economic status (*p* < 0.05) but no significant effect on class identity and affective attributions. Model 14 addresses university students with a bachelor’s degree. Sports participation had a significant contributing effect on Class identity (*p* < 0.01) and Affective attributions (*p* < 0.05). However, it had no significant effect on economic status. Social trust (*p* < 0.01) and social equity (*p* < 0.001) under the influence of sports participation significantly contributed to the social identity of this type of university student, but school level had no significant effect. Model 15 is for university students with graduate degrees. Social trust had a significant facilitating effect on affective attributions (*p* < 0.001), school level had a weakly significant facilitating effect on economic status (*p* < 0.1), and the remaining variables had no significant effect.

Under the condition of social identity, the prominence of sports participation in the social identity of university students is characterized by a decreasing school level and education level from lower to higher. In other words, the higher the school level or the higher the education level is, the fewer the social identity dimensions that are affected by sports participation, and the smaller the promotional benefits generated. This finding indicates that a synergistic nurturing pattern of sports program ideology and politics in Chinese colleges and universities has not yet formed. ① Higher-level schools have relatively strong faculty, and the nurturing of sports curriculum ideology is more systematic and scientific (Wang and Qian, 2023), prompting sports participation and students’ social identity to converge at a certain level (Shen and Zhao, 2023); however, these circumstances make it difficult for sports participation to have a significant promotional effect on university students in higher-level schools, which demonstrate a high level of sports curriculum ideology construction and a high level of students’ social identity but with initial stagnation. A breakthrough should be sought. ② Teachers in lower-level schools are relatively weak, with insufficient resources to support and safeguard civic education, resulting in the development and design of the civic elements of the curriculum failing to meet the needs of students with respect to enhancing their social identity (Wang and Qian, 2023). ③ Under the condition of education level, the influence of sports participation on the social identity of university students is in line with the real world situation. The Ministry of Education of China stipulates that physical education is mandatory for specialists and undergraduates, seeking in this way to promote students’ all-around development. In contrast, postgraduates do not face such a requirement. Therefore, sports participation has a certain promotional effect on the social identity of specialists and undergraduates but no effect on postgraduates. The possible reasons for the lack of a significant effect of sports participation on postgraduates’ social identity are as follows. On the one hand, postgraduates have already received many years of higher education. Thus, they have formed a firmly established outlook on life, values and worldview, and their social identity tends to be mature, such that there are only a few factors that can have an impact on them. On the other hand, graduate students confront complex research tasks and substantial research pressure. They do not have time to participate in sports, or they have formed a regular lifestyle and have fixed sports participation programs and schedules. Thus, sports participation cannot influence the social identity of graduate students.

## 4 Further discussion

### 4.1 The role of enhanced sports participation in the social identity of Chinese university students

#### 4.1.1 The leading effect of competitive sports should be enhanced

Leading through mass sports has been a strategic policy in sports development in China since 2023. At the welcome banquet of the World University Games in Chengdu in July, General Secretary Xi Jinping noted the importance of joining hands with the world’s youth, promoting unity through sports, gathering positive energy for the international community, jointly addressing global challenges, and carrying forward the common values of all mankind. Therefore, to give full play to the leading role of competitive sports and promote university students’ sports participation to enhance their social identity, the following 2 approaches are recommended. ① Improving the organization of university sports competitions. Multiple event organizations can be created, which can be divided into university-level games in different sports, college-level games, and joint games between different schools or colleges, with the aim of increasing the opportunities for university students to participate in sports and thus increasing the opportunities to bolster their social identities. ② Promotion of sports morality. Dynamically updating the sports competition system and promoting the construction of a sports culture over time while focusing on the cultivation of students’ sports competition ethics, referees’ professional ethics and spectators’ sports enthusiasm (Shi, 2018) as well as, ultimately, helping sports participation enhance the social identity of university students.

#### 4.1.2 Expanding the social value of sport participation

As evidenced by Models 1–3, not only does sports participation enhance the social identity of university students, but social mentality and social identity also have a certain contributing effect. Therefore, expanding the social value of sports participation is beneficial for enhancing university student social identity. Additional recommendations are as follows. ① Reducing class differences and strengthening class identity. Model 3 shows that school level and education level have different degrees of influence on university students’ class identity. Therefore, interaction and communication between university students of different levels of college and different education levels can be promoted through recreational sports or sports competitions to facilitate the transfer and common development of university students’ social identity. ② Safeguarding the demand for sports and improving economic status. Participation in sports such as badminton, swimming and fitness are restricted by venue resources, and some university students are unable to participate in sports due to economic constraints, exemplifying the serious impact of economics on social identity development through sports participation. Therefore, school resources should be fully utilized to promote the sharing of sports venues among school students to strengthen social equity and offset any economic disadvantages. ③ Expanding sports socialization and strengthening affective attributions. Creating an intelligent communication platform for sports programs would help university students in different circles establish ties with other groups, enhance social trust, and strengthen students’ affective attributions with respect to society.

### 4.2 Enhancing the effectiveness of the sports participation social mentality on the social identity of university students by deepening sports curriculum civic policies

#### 4.2.1 Improving the system of public physical education programs in colleges and universities

Models 4–9 show that, under the social mentality condition, sports participation has an overall facilitating effect on the social identity of university students. To strengthen the effectiveness of the influence of the social mentality, improving the public sports curriculum system is recommended as follows. ① Expanding the provision of sports theory elective courses in public sports curricula. On the one hand, to strengthen university students’ awareness of health development, courses such as sports psychology and exercise physiology should be taken as public sports elective courses, the theoretical knowledge of university students’ sports participation should be enriched, students’ identification with their sports participation should be promoted, and healthy sports behavior should be gradually developed. On the other hand, general public sports elective courses on sportsmanship and sports culture should be created, such as courses on the Olympic spirit and traditional Chinese sports culture, to deepen university students’ theoretical knowledge of sportsmanship and traditional sports culture and enhance their sportsmanship and social identity. ② Shaping representative public sports technical courses. Thoroughly investigating the ideological content of different sports, such as the spirit of a women’s volleyball team or table tennis diplomacy, would highlight the political connotations of sports and help spread the “general knowledge-professional-strong country” characteristics of high-quality sports and technology, for example through ideological and political courses, while promoting the implementation of sports courses in sports and technology study programs (with the strategy of strengthening the country) (Gao, 2022) and improving the level of university students’ sports and technology and the level of social acceptance.

#### 4.2.2 To promote the reform of teaching methods and the evaluation of civics in physical education programs

Under social conditions, the university civic education system has been unable to meet the needs of the times regarding the cultivation of university students’ virtues, while the endogenous elements and exogenous resources of sports course civic education are rare. For this reason, the reform of teaching methods and evaluation in sports course civic education is recommended as follows. ① A fusion teaching method should be adopted to improve the teaching effect of sports course ideology and politics. The fusion of traditional teaching methods with, for example, situational teaching methods, thematic discussion methods, classroom debate methods, or humanistic teaching methods, in teaching courses on the civics and the politics of public sports in colleges and universities could prompt students to diversify their thinking and improve their abilities in various areas, such as social trust, teamwork, and the expression of emotions, which would be an effective means to enhance the social identity of university students (Zhang et al., 2023). ② A “feedback-renewal” mechanism for the evaluation of the teaching of sports courses in the fields of ideology and politics should be established. Applying the “feedback-renewal” mechanism in the evaluation of the teaching of the ideology and politics has unique value for the creation of learned, reflective and research-oriented teacher educators and the promotion of the connotative development of the ideology and politics of the curriculum; this mechanism can be introduced by integrating professional learning as well as assessment and evaluation, subjective description and text-based observation, and classroom teaching and scale testing (Xie and Chen, 2022).

#### 4.2.3 The informatization of the teaching resources of sports courses on ideology and politics should be strengthened

① The digital transformation of teaching resources in courses on sports ideology and politics should be promoted. First, the advantages of the intelligent technology of all the elements of digitalization, full process automation, and full scene linkage to drive the rational allocation of sports curriculum resources and ideological teaching resources should be fully utilized. Second, the costs and other burdens involved in teaching ideology in the physical education curriculum through digital technology should be reduced, and an “online + offline” linkage mechanism to promote the transformation and upgrading of the teaching system of the physical education curriculum should be established. Third, artificial intelligence, the Internet of Things, and other digital technologies could be used to capture all student-related data with the aim of establishing a massive resource information database, form the sports profiles of students, and help teachers accurately grasp the dynamics of the social identity of university students while providing a reference basis for the timely adjustment of the teaching design of physical education courses (Xie et al., 2022). ② Digital technology should be used to strengthen the promotion and utilization of the teaching resources of the sports curriculum. First, teachers should be trained to rely on digital resources so as to break through traditional education boundary constraints and promote the real-time sharing and continuous flow of sports course ideological teaching resources. Second, a cross-border integration platform for different educational subjects and data network ecology of sports course ideological data should be created (Xie et al., 2022). Third, through blockchain technology, self-media technology and other digital technologies based on the actual circumstances of students’ participation in sports, students’ personal realities, and other information, intelligent and individualized teaching resources for physical education courses should be promoted to expand the scope of physical education participation to enhance the social identity of university students (Zhang and Xiangdong, 2022).

### 4.3 Differentiating among the benefits of enhancing the role of sports participation in social identity development among university students with different social identities

#### 4.3.1 Developing differentiated action strategies at the school level

The statistical results show that the effectiveness of sports participation for university students at different school levels varies. Therefore, a focus according to school level is recommended to strengthen the role of sports participation in the enhancement of the social identity of university students as follows. ① Defining the focus and action programs for different school levels. Ministry-affiliated and above universities should focus on how to promote sports participation to achieve a significant positive effect on university students’ social identity. The goal should be to make full use of physical education teaching, after-school sports, and on- and off-campus sports organizations to produce significant effects. Provincial and other universities can concentrate on 2 areas. On the one hand, efforts should be made to strengthen the role of sports participation in class identity; as Dong (2021) noted, sports participation can alleviate the expanding trend of class inequality and imprint sports participation in the life course of university students, which may help them overcome class inequality; on the other hand, universities at this level should promote sports participation in terms of economic status and affective attributions to produce a significant positive effect while fostering sports participation in developing the social identity of students. Creating a diversified, differentiated, all-round sports participation service system can prompt university students to realize the double breakthrough of economic satisfaction and an improved social identity. ② College and university sports participation should be based on institutional level and accompanied by upward learning. Ministry-level universities and above can adopt the method of integrating sports participation with national education and education on the international situation to strengthen the political literacy of university students and enhance their social identity so that they can better contribute to the development of the country and enhance their international status. Provincial and other universities, in contrast, can learn from the development experience of ministry-affiliated universities and the higher-ranking universities. In addition, they can fully explore the characteristic sports activities of their local regions and deeply integrate them into the teaching content of physical education courses and the social identity of university students to enhance the social identity of such students and make them better able to contribute to the development of their localities.

#### 4.3.2 Specific implementation pathways for different education levels should be designed

The statistical results show that the impact of sports participation on the social identity of university students differs according to academic qualifications, suggesting the following paths for targeted breakthroughs. ① Setting up specific programs of physical education for specialties and undergraduates. Public sports elective courses should be opened for the third and fourth years of the university to extend the nurturing effect of the ideological and political teaching included in such courses and to promote the sustained enhancement of the social identity of university students. ② Efforts should be made to enhance the effectiveness of sports participation on the social identity of graduate students. First, the curriculum should be optimized according to the experiences and practices of Beijing Normal University and other universities. Second, a public sports course system covering all undergraduate, master, and doctoral programs should be designed, and more public sports elective courses for postgraduates should be established to provide more opportunities for postgraduates to participate in sports and thus strengthen their social identity. Third, a structured pushback mechanism, based mainly on, e.g., prizes, merit, or credit points, should be developed to establish sports participation as a necessary factor for “eligibility” and to comprehensively formulate sports participation in terms of projects, duration, and physical fitness factors that must be developed. Fourth, school sports games and other events should be used to enhance social identity. Finally, with school sports games and other kinds of sports competitions as important tools, we should establish competitions that address the characteristics of the physical and mental development of postgraduates, guide postgraduates to actively participate in sports, promote the development of healthy sports behaviors, and enhance the physical and mental health as well as social identity of postgraduates.

## 5 Conclusion

Comprehensively promoting the sports participation of Chinese university students to enhance their social identity is an important measure for delivering high-quality talent to modern China’s economy and society, implementing China’s national strategy and accelerating the promotion of the socialist economic system with Chinese characteristics. From a holistic perspective, sports participation can enhance the social identity of university students in all aspects, and the benefits are positively related to the frequency of sports participation. Under the condition of social mentality, sports participation has a promoting effect on the social identity of university students in general and a nonsignificant effect only on certain dimensions. Under the condition of social identity, the significance of sports participation on the social identity of university students is characterized by a decreasing effect according to school level and education level from low to high. On the basis of the actual situation in China and our empirical results, we propose a targeted approach to using sports participation to enhance the social identity of Chinese university students as follows. ① Strengthening the contribution of sports participation to the social identity of Chinese university students to enhance the leading effect of competitive sports and expand the social value of sports participation. ② Enhancing the effectiveness of sports participation on the social identity of university students under social mentality conditions by improving the design of sports curriculum civics courses, improving the public sports curriculum system in universities, promoting the reform of sports curriculum civics teaching methods and evaluation, and strengthening the informatization of sports curriculum civics teaching resources. ③ Differentiating among the benefits of enhancing the role of sports participation in social identity among university students with different social identities, including by developing differentiated action strategies according to school level and designing specific implementation paths for different education levels.

## Data Availability

The datasets presented in this study can be found in online repositories. The names of the repository/repositories and accession number(s) can be found at: http://cgss.ruc.edu.cn.
